# Sample Entropy of sEMG Signals at Different Stages of Rectal Cancer Treatment

**DOI:** 10.3390/e20110863

**Published:** 2018-11-09

**Authors:** Paulina Trybek, Michal Nowakowski, Jerzy Salowka, Jakub Spiechowicz, Lukasz Machura

**Affiliations:** 1 Division of Computational Physics and Electronics, Institute of Physics, Silesian Centre for Education and Interdisciplinary Research, University of Silesia in Katowice, 40007 Katowice, Poland; 2 Department of General Surgery and Multiorgan Trauma, Jagiellonian University Medical College, 30048 Krakow, Poland; 3 Department of Surgery, Stanley Dudrick Memorial Hospital, 32050 Skawina, Poland; 4 Department of Theoretical Physics, Institute of Physics, Silesian Centre for Education and Interdisciplinary Research, University of Silesia in Katowice, 40007 Katowice, Poland

**Keywords:** surface electromyography, colorectal cancer, sample entropy, multiscale entropy

## Abstract

Information theory provides a spectrum of nonlinear methods capable of grasping an internal structure of a signal together with an insight into its complex nature. In this work, we discuss the usefulness of the selected entropy techniques for a description of the information carried by the surface electromyography signals during colorectal cancer treatment. The electrical activity of the external anal sphincter can serve as a potential source of knowledge of the actual state of the patient who underwent a common surgery for rectal cancer in the form of anterior or lower anterior resection. The calculation of Sample entropy parameters has been extended to multiple time scales in terms of the Multiscale Sample Entropy. The specific values of the entropy measures and their dependence on the time scales were analyzed with regard to the time elapsed since the operation, the type of surgical treatment and also the different depths of the rectum canal. The Mann–Whitney U test and Anova Friedman statistics indicate the statistically significant differences among all of stages of treatment and for all consecutive depths of rectum area for the estimated Sample Entropy. The further analysis at the multiple time scales signify the substantial differences among compared stages of treatment in the group of patients who underwent the lower anterior resection.

## 1. Introduction

The comprehensive knowledge about the information hidden in the complex surface electromyographic (sEMG) signals of the external anal sphincter (EAS) could significantly contribute to the proper assessment of the activity of this specific muscle group in the context of patients after multimodal rectal cancer therapy. Colorectal cancer (CRC) is one of the most frequent cancers worldwide and nowadays represents a significant part of the major public health problems [1]. Increasing morbidity and mortality rates indicate a rising global burden of CRC [2]. The latest predictions for 2030 estimate approximately 2.2 million new cases per year [3]. The standards of patient care require complex multimodal treatment composed of surgery, irradiation, and chemotherapy. The medical protocol is strongly dependent on type, localization and the stage of CRC. Especially the first two mentioned treatment modalities can have a significant impact on the long term quality of life after the therapy due to their side effects. Those of special importance include stool and gas control and can range from minor gas leak to complete stool incontinence or evacuation difficulties. Frequency of those problems earned them even a separate name and are often referred to as (Low) Anterior Resection Syndrome (LARS) [4].

It is also documented that the surgery, especially the level of anastomosis in conjunction with neoadjuvant radiotherapy, could increase the risk of postoperative complications associated with fecal incontinence [5]. Despite many exhaustive reports about LARS [6,7], it is still not absolutely clear what kind of pathophysiologic mechanisms are most responsible for the postoperative dysfunction of the EAS muscle group. There are some suggestions that innervation injuries might have a relevant contribution to the multifractional LARS etiology [8].

The distribution of innervation zones (IZ) shows a large discrepancy in the studied groups and relatively high level of individual patient asymmetry [9,10]. Thus, the difficulties in proper cognition of the main source of LARS are also dictated by the significant impact of intersubject variability. The other issues of great importance concern the different locations of anastomosis in the rectal area regarding the proximity of the sphincter muscles or the destructive effect of radiation. All of these factors lead to the conclusion that the sphincter-sparing procedures require the thorough diagnostic tests of EAS neuromuscular system at every stage of the treatment process to be able to properly choose treatment regimens and assess risk factors. Among the applied techniques for monitoring the activity of EAS, considerable attention has been paid to the methods of electromyography (EMG).

Previous studies characterize the coaxial needle technique as an effective tool for investigating the neural control of EAS in the patient with defecation disorders [11]. However, due to some limitations of this method, mainly caused by its invasive character and technical difficulties related to a low repeatability of measurements (sampling error due to the placement of needle electrodes), the surface electromyography (sEMG) as a non-invasive equivalent has gained a wide range of application in this field [12,13,14,15,16]. The exhaustive report of available techniques for acquiring the sEMG data from the external anal sphincter was presented by Merletti in [17]. The continuous progress in the construction of measurement devices allows the gathering of new valuable information about the EAS motor units such as the precise localization of the active innervation zones. Despite these experimental successes, the literature still lacks the comprehensive theoretical characterization of raw sEMG in this specific clinical context. sEMG signals always represent complex nature with low signal to noise ratio [18]. The ability to monitor the whole group of motor units from some distance entails, in turn, a negative cross-talk effect due to the impact of the neighboring muscle activity. The tissue characteristics or the noise generated by external devices are among common factors which intensively influence the morphology of the signal wave.

To get a more profound insight into information hidden in sEMG, the use of proper analytical methods which can cope with the complex character of the examined phenomena is required [19]. The information theory with the special emphasis on the entropy-based techniques has become one of the very promising branches among the variety of algorithms used in biomedical signal processing [20,21,22,23]. Under normal healthy conditions, physiological systems are characterized by high dynamical complexity which is conditioned by their ability of quick adaptation to an incessantly changing environment. The loss of such complexity is often related to the pathological state [24].

The concept of entropy for a characterization of the measured data was first proposed in 1948 by Shannon in the form of the logarithmic dependence on a probability density function [25]. Further studies in this field resulted in the development of several forms of entropy measures, from a notion of the Spectral Entropy, through the more advanced techniques such as Approximate Entropy (AE) or its updated version Sample Entropy (SampEn) up to the Fuzzy Entropy presented by Chen et al. in 2007 [26].

A key limitation of these techniques is that they do not take into account multiple time scales. Biosignals often exhibit different behaviors depending on an actual scale. Nonlinearity, long memory or sensitivity to small disturbances are among the phenomena for which the description limited to a single time scale may not be sufficient. Although there exists a variety of entropy measures, the most widely used method in the context of a physiological signal’s dynamics is Multiscale Entropy algorithm proposed by Costa et al. [27,28,29,30,31]. In recent years, several authors proposed an application of multiscale entropy and proved the method as a successive one for biomedical data analysis [32,33]. One of the applications includes the description of the sEMG, i.e., the activity of the urethral sphincter function [34] or a classification of the muscular disorders [35]. The aim of this work was to contrast the signals recorded at the different stages of rectal cancer treatment through the extensive analysis based on entropy parameters. Both the specific values of the entropy measures and their dependencies on the time scales were analyzed due to factors such as the type of surgical treatment and the time of the recovery after the operation. In addition, the contraction and relaxation states at the different anatomical levels of the signal acquisition were considered separately.

## 2. Methods

### 2.1. Sample Entropy

The Sample Entropy (SampEn) represents the updated version of that developed by Pincus in 1991 Approximate Entropy (ApEn) [36]. There are several approaches for obtaining these entropy features. A brief description of the SampEn algorithm used in this work is presented below. For more details, see [37,38].

The calculation of SampEn for the time series ${\left\{x_{i}\right\}}_{i=1}^{N}$ which consists of *N* data points requires a prior determination of the two parameters: (i) the embedding dimension *m* which characterizes the length of vectors to compare; and (ii) the tolerance threshold *r* referred to as a similarity criterion or the distance threshold for two template vectors. The latter is usually chosen from the range between 10% and 20% of the standard deviation σ of the signal’s amplitudes [39]. In the following, the values of m=4 and r=0.2σ have been used.

The procedure starts with the definition of a set of vectors $U_{m}\left(i\right)$ that represent *m* consecutive values of series, starting with the *i*th point

(1) $$U_{m}\left(i\right)=\{x_{i},x_{i+1},\cdots ,x_{i+m-1}\},\;1\leq i\leq N-m+1$$

Next, the Euclidean distance between the $U_{m}\left(i\right)$ and $U_{m}\left(j\right)$ is estimated as the absolute maximum difference between their scalar components:

(2) $$d\left[U_{m}\left(i\right),U_{m}\left(j\right)\right]=\max_{k=0,\cdots ,m-1}(|x\left(i+k\right)-x\left(j+k\right)|)$$

In the next step, the probability $C_{i}^{m}\left(r\right)$ that any $U_{m}\left(i\right)$ vector is close to $U_{m}\left(j\right)$ is determined. The $n_{i}^{m}\left(r\right)$ stands for a number of $U_{m}\left(j\right)$ vectors (1≤j≤N−m, j≠i) that do not exceed the accepted tolerance threshold *r* i.e., $d[U_{m}\left(i\right),U_{m}\left(j\right)]\leq r$.

(3) $$C_{i}^{m}\left(r\right)=\frac{n_{i}^{m}\left(r\right)}{N-m}$$

This value is averaged over all possible pattern vectors $U_{m}\left(i\right)$ to estimate the probability $C^{m}\left(r\right)$ that any two vectors are within *r* of each other

(4) $$C^{m}\left(r\right)=\frac{1}{N-m+1}\sum_{i=1}^{N-m+1}C_{i}^{m}\left(r\right)$$

Finally, the SampEn is negative logarithm of the conditional probability that two sequences similar for *m* points remain similar for the m+1 points.

(5) $$SampEn\left(m,r,N\right)=-\ln\left[\frac{C^{m+1}\left(r\right)}{C^{m}\left(r\right)}\right]$$

For the above calculations, j≠i, which means that self matches are not taken into account as in the case of earlier ApEn.

### 2.2. Multiscale Entropy

The estimation of Multiscale Entropy (MSE) consists of two main steps. The first part implements the coarse-graining procedure of resampling the series to explore different time scales of a signal [40]. The multiple coarse-grained time series are obtained by averaging the data points in each of the non-overlapping windows with the increasing length. The procedure for the calculation of each of the coarse-grained series for the consecutive scale factors τ is given by

(6) $$y_{j}^{\tau }=\frac{1}{\tau }\sum_{i=(j-1)\tau +1}^{j\tau }x_{i},\;1\leq j\leq \frac{N}{\tau }$$

The second stage concerns the calculation of the sample entropy which was just presented in the previous Section 2.1. For each $y_{j}^{\tau }$ series, the value of SampEn is calculated and plotted as a function of τ resulting in the MSE curves.

## 3. Material

### 3.1. sEMG Signal Source

**Figure 1:**
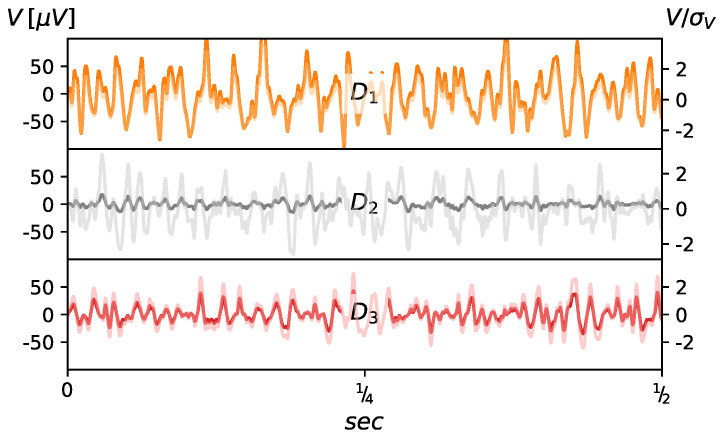
The exemplary original (lhs scale, normal lines) and normalized (rhs scale, translucent lines) EMG data registered from the one of 16 channels at three different stages of treatment.

The examined time series were recorded at three stages of treatment, before the surgical procedure ($D_{1}$) and on two occasions in the postoperative period ($D_{2}$ and $D_{3}$): one month after surgery $D_{2}$ and at one year $D_{3}$. The exemplary raw and normalized EMG data are presented in Figure 1. Normalization was performed with respect to the standard deviation, i.e., $\hat{V}=V/\sigma_{V}$. The data acquisition system consists of the anal probe developed at the Laboratory of Engineering of Neuromuscular System and Motor Rehabilitation, Politechnico di Torino in collaboration with the OT-Bioelettronica company. The signals were acquired from the three rings of 16 silver/silver oxide bar electrodes (1×10 mm) placed parallel to the long axis of the probe. Inter-ring distance was 8 mm and that allowed for signal recording at approximate depths of 1, 3 and 5 cm from the anal verge. The probe worked in conjunction with the standard PC over 12 bit NI DAQ MIO16 E-10 transducer (National Instruments, Austin, TX, USA). The measurement protocol included: 1 min of relaxation, and three 10 s long recordings at rest for each depth, 1 min relaxation, and then three 10 s long recordings at maximum voluntary contraction (MVC) for each depth with additional 1 min breaks in between. Each single 10 s long measurement with the sampling frequency of 2048 Hz gave a series composed of 20,480 data points. Low and high pass filters were used at 10 and 500 Hz, respectively. This resulted in typical 3 dB bandwidth for the Analog-to-Digital Converter.

### 3.2. Patients

The study group included 20 subjects, 7 female, age range 46–71 (average 57.14 ± 9.59 years) and 13 male, age range 48–85 (average 69.6 ± 10.04 years), diagnosed with a rectal cancer and qualified for surgery. All underwent open, transabdominal resection. Based on the distance of colorectal stapled anastomosis, the study group of patients was divided according to the decision of the operating surgeon. For surgeons to make decision on the type of the procedure (AR vs. LAR in this case), a localization of the tumor is crucial. It is common to decide that tumors localized in the upper third of the rectum require AR, those in middle portion LAR and those lower than that in some cases need LAR, ultra low LAR or abdomino-perineal resection. The patients with anastomosis at or below 6 cm from the dentate line were included in the Low Anterior Resection (LAR) group. Those with higher anastomosis were included to the Anterior Resection (AR) group. Indirectly, a level of anastomosis implies also the extent of mesorectal excision with all patients in LAR group undergoing Total Mesorectal Excision while in the case of AR group mesorectum was excised minimum 5 cm below the lower margin of tumor. For the detailed information on the surgical landmarks of rectum, see [41]. The group of patients is equally distributed with respect to the type of surgery: 10 subjects with AR (average years 62.4 ± 11.14) and 10 with LAR (average years 67.3 ± 9.97). The LAR group includes eight males and two females, and except from one case, all the patients underwent the neoadjuvant radiotherapy (5×5 Gy for the total of 25 Gy). The anterior resection group consists of five male and five female subjects and none of them received neoadjuvant radiotherapy. In both groups, TNM classification of patients was similar. In AR group, there were six patients with T2 tumor and four patients with T3 tumor. In LAR group, we had five patients with T2 and five with T3 tumors. In both cases. resection was carried out along predefined planes and for the same localization T2 and T3 tumors underwent the same resection. Regarding lymph node involvement, two patients in AR group and three patients in LAR group were N1. All others were N0. Regarding chemotherapy, patients with positive lymph nodes received adjuvant chemotherapy.

## 4. Results

### 4.1. Choice of Embedding Dimension Parameter

**Figure 2:**
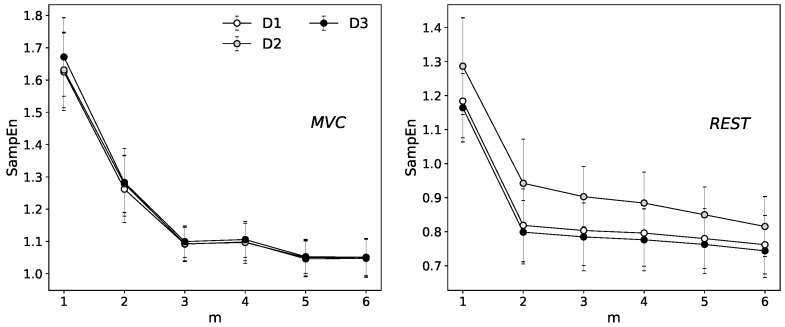
The Sample entropy calculated for the relaxation and maximum contraction state at the different embedding dimension setting: (**left**) the maximum contraction state; and (**right**) the relaxation state. The results are presented as an average of the 16 channels of selected state $D_{1}$.

The standard protocol for the proper evaluation of motor units activity of the EAS muscle group recommends a minimum sampling frequency of about 2 kHz [42,43] and our data meet that restriction. However, the power spectra density estimation indicates the highest oscillations around 500 Hz (for details see [23]). To eliminate the potential effect of overestimation of SampEn through the comparing of the segments that consist of points with the same contribution to the signal, in other words, to avoid the situation that the four adjacent samples selected to form patterns we decided to choose m=4. The effect of stabilization of SampEn function along with the increase of embedding dimension is presented in Figure 2.

### 4.2. Single Scale Entropy

**Figure 3:**
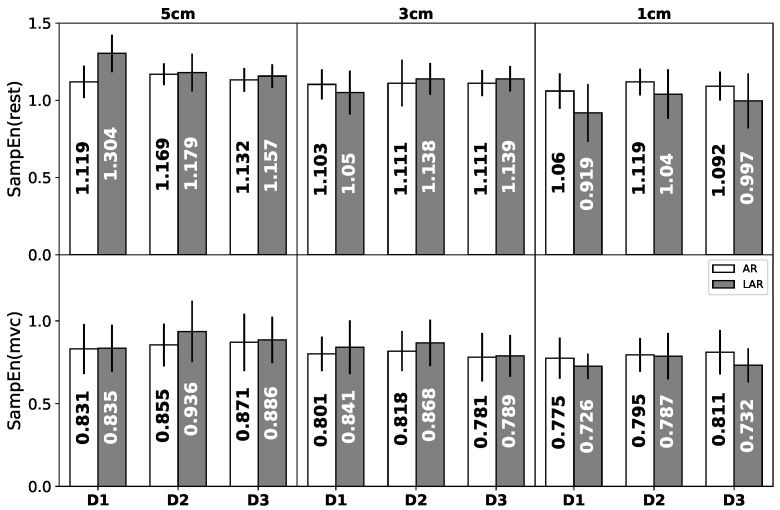
The average values of sample entropy calculated for patients with AR (white) and LAR(gray) at different stages of treatment ($D_{1}$–$D_{3}$). The upper graphs represent, respectively, different depths of relaxation states. The lower charts are assigned to the maximum voluntary contraction.

The results of SampEn are presented in Figure 3. There is a very clear division between the contrary states of EAS muscle tension.

The significantly greater values of SampEn for the relaxation state are partially justified by the concept of entropy as a measure of the diversity of the available states in the system. The reduction of these states is a consequence of the contraction phenomenon itself. During the propagation of the action potential within the functional motor units, the specific direction of the process is dominated which automatically entails the decrease of the number of possible states that the system can choose. The statistically significant differences calculated via Wilcoxon signed rank test at the selected significance level α=0.05 were identified between the relaxation and maximum voluntary contraction in all the individual stages of treatment (D1–D3) and the respective depth of rectum canal (5 cm–1 cm). Interesting results concern the decrease in the mean values of sample entropy along with the rectum canal depth. Comparing the signals registered at 5 cm and 1 cm of depth in the case of contraction, the lower values of SampEn are assigned to the signals acquired in the immediate vicinity of a sphincter. The most visible differences between AR and LAR group seem to characterize the relaxation state at 1 cm of depth. At this specific level of the rectum, the AR group is characterized by the higher values of SampEn. The analogical tendency is visible for the maximum voluntary contraction.

### 4.3. Multiscale Entropy (MSE)

**Figure 4:**
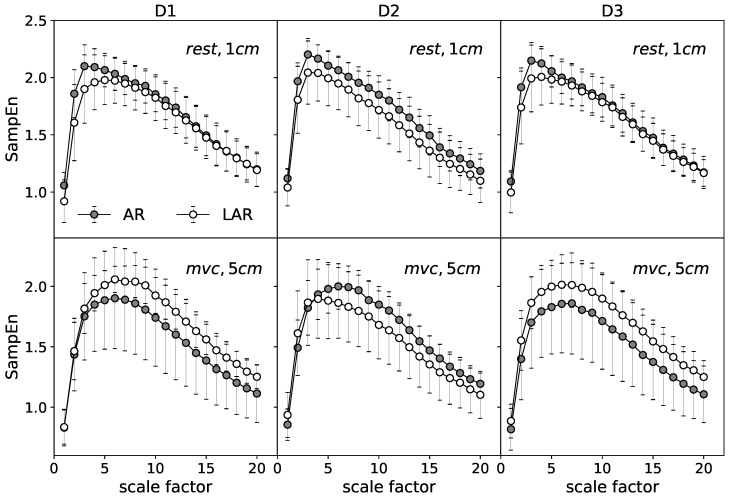
The mean MSE entropy curve obtained for the selected cases: comparison of the group with AR and LAR for each of the treatment stages.

The in-depth description of the examined data is given by SampEn calculated over multiple timescales. Figure 4 gives an example of mean MSE curves for the selected representative stages. Each point presents an average of 160 values of SampEn (16 signals per one subject) calculated for the respective coarse grained series at the consecutive scales τ⊂[1,20]. The differences of the MSE analysis between AR and LAR groups for the selected cases are illustrated. The upper graphs represent the relaxation state at 1 cm of anal canal depth. The lower panels are assigned to the maximum voluntary contraction recorded at 5 cm. For both the relaxation and MVC, $D_{2}$ significantly stands out from the other stages and there are no visible differences between the most distant stages of treatment $D_{1}$ and $D_{3}$. The mean MSE curves of the state before surgery ($D_{1}$) and one year after operation ($D_{3}$) retain almost identical for the compared groups AR and LAR. Considering the different stages of muscle tension individually, the curves that illustrate the group with LAR are located respectively lower in the case of the relaxation state. The most visible differences identified at all ranges of scale occur one month after the surgery ($D_{2}$).

**Figure 5:**
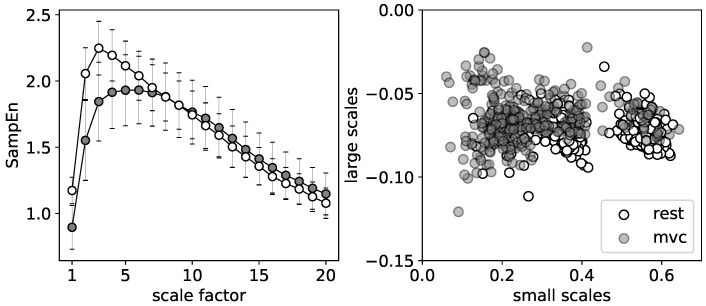
The comparison of relaxation and MVC stage (an example calculated for both surgery group at 5 cm of depth one month after operation: (**left**) MSE curves; and (**right**) a point representation of MSE curves.

Quite a different result is found for the MVC where the AR group is characterized by the reduced values of entropy in comparison to the LAR for $D_{1}$ and $D_{3}$ stages. Only the stage $D_{2}$ one month after surgery expresses higher values of SampEn at the all scaling range. In this case, the group of patients with LAR is represented by the lower values of SampEn for larger time scales. In general, the shape of curves assigned to the MVC and relaxation states have similar character. The rapid increase up to a certain maximum value at the relatively small scaling range and the monotonic decrease for the large scale factors. The observed differences are mainly manifested by the location of the maxima $\tau_{max}$. At relaxation state, the highest value of SampEn is identified around $\tau_{max}=3$, whereas for the contraction the maxima of MSE curves are shifted to the higher values of $\tau_{max}=6$. The MVC curves are also smoother around the maximum than in the case of relaxation. For better visualization of the differences between those contrary stages of muscle tension an example of MSE curves of relaxation and MVC state are presented together in Figure 5 (left).

In addition, Figure 5 (right) presents a point representation of respective curves. Each point of the individual scatter plot is characterized by its respective coordinates. The abscissa represents the slope coefficients of the linear fit of the MSE curves for the small scales, i.e., the ranges of scales between 1 and $\tau_{max}$. Accordingly, the ordinate is assigned to the slope coefficients of the linear fit for the large scales, i.e., $\tau >\tau_{max}$. Contrary to the relaxation in the case of MVC, the majority of points aggregate at the lower values of $\tau <\tau_{max}$. The slopes of the fit for the large scales are similar for both stages.

Considering the different stages of treatment, the middle case, $D_{2}$, appears to possess the highest variability among all presented cases. For this reason, the comparison of the stages before and after surgery individually for the LAR and AR groups needs to be addressed. In the following, the $D_{3}$ stage is omitted for the sake of the clarity of presentation.

**Figure 6:**
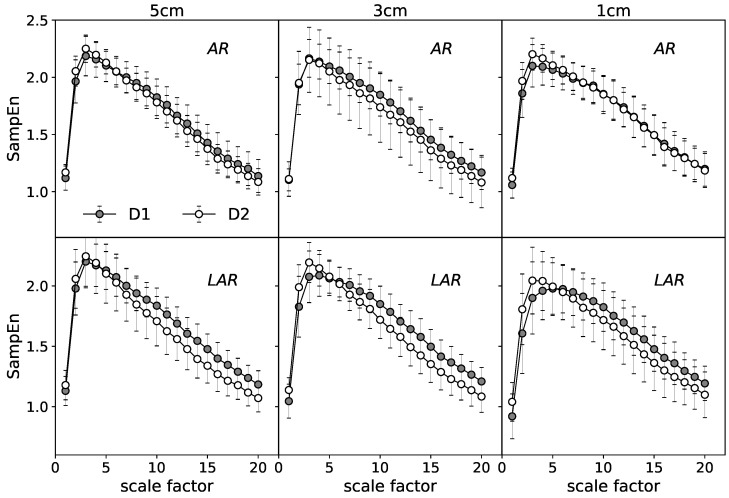
The mean MSE entropy curves obtained for the relaxation state: comparison of stages D1–D2 in the group with AR and LAR.

**Figure 7:**
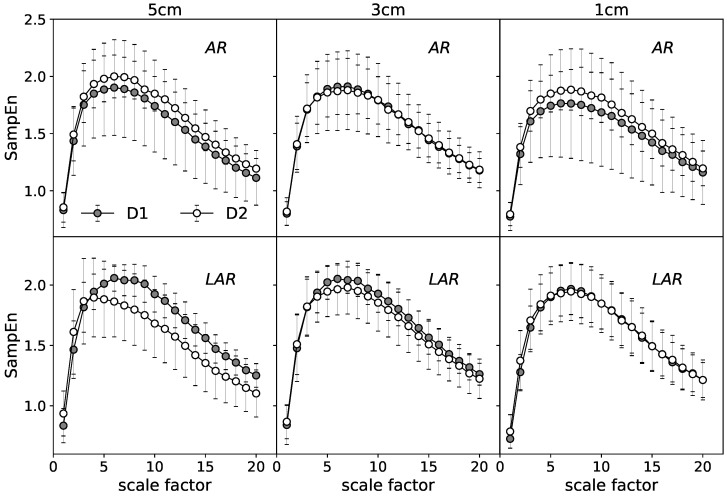
The mean MSE entropy curves obtained for the MVC state: comparison of stages D1–D2 in the group with AR and LAR.

Figure 6 present the results at the relaxation state in the form of mean MSE curves. The upper graphs characterize the AR group. The lower panels are their equivalent for the patients with LAR. The analog set of results is given for the MVC (Figure 7).

A general comparison of the mean MSE curves characterizing the distinct states of EAS tension (Figure 6 vs. Figure 7) indicates the more visible differences between compared stages for the phase of relaxation. For this stage, patients with LAR are characterized by the larger differences between $D_{1}$ and $D_{2}$ at all registered depths of rectum canal. The values of SampEn are, respectively, lower for the $D_{2}$ over all considered range of scales. The more pronounced differences seem to refer to the LAR group. The contraction phase indicate visible differences between respective MSE curves at 5 cm of depth in the LAR group. In contrast to the AR group, D2 stage is characterized by the lower SampEn values at the whole scaling range.

## 5. Statistics

The normality test of the entropy functions calculated via Shapiro–Wilk formula does not allow us to confirm the hypothesis about the normal distribution for the majority of analyzed cases. Thus, to characterize the differences between compared stages the non-parametric statistical tests were used. The comparison between AR and LAR group are presented in Table 1. It consists of the results of the Mann–Whitney U test (the non-parametric equivalent of t-student statistics for the independent samples). The *p*-values, calculated at the selected significance level (α=0.05), are presented for each entropy measures. The statistically significant differences are featured in bold. The individual stages of treatment (D1–D3) together with the respective depths of rectum canal (5 cm–1 cm) are taken into consideration. Table 1 sets together results of single scale sample entropy and MSE considered as an average values of SampEn at all scaling range. The larger divergence between the AR and LAR groups is observed for the relaxation state at 1 and 5 cm of depth. For the multi-SampEn comparison, the MVC stage shows differentiation at the all compared states.

Next, the statistical differences within the individual groups were also investigated. The results of Anova Friedman statistics, a widely known non-parametric analog of the one-factor analysis of variance for the repeated measurements, indicates a statistically significant difference (p<0.05) between the consecutive stages of treatment at the individual depths of a rectum canal as well as the corresponding depths for the separate treatment periods. The respective differences were identified for both single scale SampEn and MSE values. The single scale SampEn results indicate statistically significant differences between all comparing stages (p<0.05). The full Friedman test characteristic of MSE values are presented in Table 2 and Table 3, respectively. Only two exceptions do not allow us to reject the null hypothesis about the lack of statistically significant differences among the compared stages. The state D2 at relaxation in the case of LAR group and D1 at MVC registered from the patient with AR are not diversified due to the depth of anal canal (see Table 3).

## 6. Discussion

This work presents an application of the selected entropy-based techniques to study the variability of information within sEMG signals at the different stages of the rectal cancer treatment. To distinguish the groups of patients due to the type of surgery as well as to compare of signals recorded at the various postoperative periods, both single and multiscale sample entropy algorithms were implemented. The statistically significant differences identified among all of the compared stages of treatment ($D_{1}$–$D_{3}$) and the different depths of rectum canal (1 cm–5 cm) were revealed by the Sample Entropy.

Definitely the most valuable information is provided by the analysis of SampEn over multiple time scales. Through the interpretation of the mean MSE curves the stages of the most visible differences between AR and LAR groups were identified one month after operation $D_{2}$ for, respectively, 1 cm depth at the rest and 5 cm depth in the case of the MVC. That corresponds well to the clinical data as the LARS syndrome has its peak severity right after surgery with the diminishing frequency and severity months after the treatment [44,45]. In addition, since the amplitude of the sEMG signal depends on the distance between its source and the electrode and that distance is the smallest for superficial part of an external anal sphincter in resting conditions, the sEMG signal is almost always the strongest in the most external recordings. In maximum contraction conditions, when the amplitude of the signal rises, the probability that the signal from deeper parts of the muscle will be sufficiently represented in the recording also rises.

It is shown that the information carried out by the sEMG signals measured one year after the surgery $D_{3}$ returns to the state of the first examination $D_{1}$ for the selected cases. This situation is also confirmed in a clinical practice since, for those patients who improve, the return of a normal function happens within the period of the first year [46,47]. Probability of later recovery is typically very small. The data acquired one month after the operation $D_{2}$ are also characterized by the lower values of SampEn for the majority of cases for the large time scales in the LAR group which can indicate to the greater impact of adverse phenomena associated with postoperative side effects in this very group of patients. Study of the stages before and one month after surgery in patients with AR and LAR individually show more visible differences for the latter group with the decrease of SampEn values at the $D_{2}$ stage. Statistically significant differences are observed among almost all of the compared stages of treatment as well as the various rectum canal depths in both AR and LAR groups. Nonetheless, the group of patients who underwent the LAR is definitely more diversified based on the MSE. That may indicate different degree of injury to a neuromuscular system resulting from a multimodal treatment of those patients. Correlation of those changes with results of functional testing are lacking, thus making further conclusions speculatory.

The main limitations of this study are due to the problem of inter-subject variability. The large diversity in distribution of EAS innervation zones, mainly caused by the high level of the individual asymmetries, significantly affects the differences between the compared groups. This phenomenon is further strengthened by the diversity of signals within a single subject. The values of the entropy for the time series detected at one of three separated rings, which consist of 16 channels each, indicate relatively high variability over these channels. That discrepancy consists of many factors including the concept of *weighted innervation zones*. Some of the innervation zones may have a greater importance than the others because of the different sizes of motor units [17]. We are not able to specify the series that characterize such dominant zones, therefore the results are averaged over all channels. Despite the relatively small values of standard deviations, an effect of inter-channels variability significantly influences the final results.

## Figures and Tables

**Table 1:** The comparison of AR and LAR group: results of the non-parametric Mann–Whitney U test calculated for entropy parameters. The test was estimated for the MVC and the relaxation states at 1, 3 and 5 cm depth of the anal canal and consecutive stages of treatment: D1–D3.

	SampEn			MSE (All Scales)		
rest	5	3	1	5	3	1
$D_{1}$	**0.050**	**0.001**	**0.000**	**0.027**	0.579	**0.000**
$D_{2}$	**0.004**	0.062	**0.000**	**0.007**	0.181	**0.000**
$D_{3}$	**0.000**	**0.001**	**0.000**	0.901	0.245	**0.000**
MVC	5	3	1	5	3	1
$D_{1}$	0.602	0.228	**0.000**	**0.000**	**0.000**	**0.000**
$D_{2}$	**0.000**	**0.004**	0.464	**0.000**	**0.000**	**0.000**
$D_{3}$	**0.000**	**0.001**	**0.000**	**0.000**	**0.000**	**0.000**

**Table 2:** The comparison of treatment stages at consecutive depth of rectum canal: full statistics of the non-parametric Friedman test calculated for SampEn entropy at all scaling range.

	AR				LAR		
rest	5	3	1	5	3	1
Anova $\chi^{2}$	57.07	106.64	86.14	413.45	384.08	43.51
Kendall coeff.	0.009	0.017	0.014	0.065	0.060	0.007
*p*-value	**0.000**	**0.000**	**0.000**	**0.000**	**0.000**	**0.000**
MVC	5	3	1	5	3	1
Anova $\chi^{2}$	110.17	55.77	261.24	517.23	302.40	55.61
Kendall coeff.	0.017	0.009	0.408	0.081	0.047	0.009
*p*-value	**0.000**	**0.001**	**0.000**	**0.000**	**0.000**	**0.000**

**Table 3:** The comparison of different depth of rectum canal at consecutive stages of treatment: full statistics of the non-parametric Friedman test calculated for SampEn entropy at all scaling range.

	AR			LAR		
rest	D1	D2	D3	D1	D2	D3
Anova $\chi^{2}$	24.05	173.95	8.612	110.88	2.974	28.67
Kendall coeff.	0.004	0.027	0.001	0.017	0.46 × $10^{-3}$	0.004
*p*-value	**0.000**	**0.000**	**0.013**	**0.000**	0.226	**0.000**
MVC	5	3	1	5	3	1
Anova $\chi^{2}$	0.645	96.11	294.97	350.50	351.96	447.34
Kendall coeff.	0.1 × $10^{-3}$	0.015	0.046	0.055	0.055	0.070
*p*-value	0.724	**0.001**	**0.000**	**0.000**	**0.000**	**0.000**

## Abbreviations

The following abbreviations are used in this manuscript:

EAS	external anal sphincter
sEMG	surface electromyography
AR	anterior resection
LAR	lower anterior resection
MVC	maximum voluntary contraction
